# Attitudes and referral patterns of lung cancer specialists in Europe to Specialized Palliative Care (SPC) and the practice of Early Palliative Care (EPC)

**DOI:** 10.1186/1472-684X-13-59

**Published:** 2014-12-16

**Authors:** Haris Charalambous, Athanasios Pallis, Baktiar Hasan, Mary O’Brien

**Affiliations:** Bank of Cyprus Oncology Centre, Nicosia, Cyprus; EORTC Headquarters, Brussels, Belgium; Royal Marsden Hospital, London, UK; EORTC Lung Cancer Group, Brussels, Belgium

**Keywords:** Lung cancer specialists, Referrals, Attitudes, Specialized palliative care

## Abstract

**Purpose:**

To examine availability of Palliative Care (PC) services and referral patterns of European Lung cancer specialists to PC.

**Methods:**

All members of the EORTC Lung Cancer Group (LCG) were asked via email to participate in an on-line survey.

**Results:**

50 out of 170 (29.4%) replied: 24 medical oncologists, 14 radiation/clinical oncologists, 11 pulmonologists and 1 thoracic surgeon. All but two of respondents (96%) had access to at least one component of PC services. In terms of referral of patients to PC almost 75% of respondents would refer most of their patients when there were no treatment options or at the end of life, while only 22% would refer patients at earlier stages of disease. Barriers for referral to PC were negative attitudes of patients to PC (26%), lack of availability of PC services (20%), lack of expertise of PC physicians(18%), the belief that referral to PC signifies abandoning patients (8%), and that PC specialists discourage active oncological therapy (8%). Whilst most of the respondents expressed positive attitudes, 12-22% had overtly negative attitudes towards PC. Seventy-eight (78%) of respondents expressed an interest to participate in a trial of early PC (EPC).

**Conclusion:**

Despite good availability of SPC services at institutions of members of the EORTC LCG, and most respondents expressing positive attitudes towards PC, their practice involved referral of patients to PC late in the disease trajectory, hence Lung Cancer specialists in Europe have not adopted the practice of EPC concurrent with active oncological care.

**Electronic supplementary material:**

The online version of this article (doi:10.1186/1472-684X-13-59) contains supplementary material, which is available to authorized users.

## Background

According to a recent statement from the American Society of Clinical Oncology (ASCO), Standard Oncology Care (SOC) today remains focused on disease directed therapy, often without realistic conversations about prognosis, the potential benefits and limitations of disease-directed therapy, and the potential role of Palliative Care (PC) [1]. PC on the other hand, has a focus on providing “active, holistic care of patients with advanced, progressive illness, including management of pain and other symptoms, where provision of psychological, social and spiritual support is paramount” [2]. Furthermore the goal of PC is “achievement of the best quality of life for patients and their families”, whilst “many aspects of PC are also applicable earlier in the course of the illness in conjunction with other treatments” [2].

Objective evidence of the benefits of early integration of PC concurrent with SOC comes from a recent randomized clinical study by Temel et al [3], who randomized patients with advanced metastatic lung cancer to SOC combined with Early Palliative Care (EPC) versus SOC alone. The EPC used in this study was an active, intensive, at least monthly visit with a palliative care consultant or specialist nurse. Patients assigned EPC had a better quality of life, with fewer patients experiencing depressive symptoms than in the SOC group [3]. Median survival was longer among patients receiving EPC, despite the fact that fewer patients in the EPC group received aggressive end-of-life care [3]. In response to the Temel study, Smith et al [4] in an ASCO Provisional Clinical Opinion (PCO) reviewed seven randomized studies examining the role of EPC including the Temel study. The ASCO PCO recognized the importance and benefits of EPC in terms of improvement of patients’ symptoms, quality of life and satisfaction, whilst reducing caregiver’s burden, and made a firm recommendation that EPC should be considered early in the course of illness of all patients with advanced or metastatic cancer [4].

In view of this evidence, this questionnaire was devised to capture valid descriptive data regarding availability of PC services in the respective European Centres, where members of the European Organization of Research and Treatment of Cancer (EORTC) Lung Cancer Group (LCG) work, as well as attitudes and referral patterns of Lung Cancer (LC) specialists to PC.

## Methods

### Survey sample/procedures

All members of the EORTC LCG were asked via email to participate in an on-line survey to examine attitudes and referral patterns to PC. All participants received a group email introducing and explaining the study and containing a link to the survey. No incentives were offered for participation. A reminder was sent after approximately 4 and 8 weeks and the survey website was closed after 10 weeks.

Neither informed consent nor ethics committee approval was deemed necessary for this study, given that this was a survey of health care specialists and did not involve research with human material or human data.

All statistical analyses, graphs and tables were performed using SAS^©^ Version 9.4 of the SAS System for Windows [5]. Correlations between categorical variables were assessed by Fisher exact test. This test is similar to Chi-squared test but it is more robust to the existence of empty cells or small sample size. Nevertheless, due to a relatively low sample size in this study and with a number of conducted tests, results of statistical tests should be viewed as exploratory in nature and hence should be interpreted with caution.

### Questionnaire

The questionnaire was developed on the basis of existing literature from similar surveys undertaken in the past [6–9], and is available as Additional file 1.

## Results

### Participant characteristics

50 out of 170 (29.4%) EORTC LCG members from 40 out of 101 different EORTC centres, and from 14 of 21 countries replied.

There were 24 (48%) medical oncologists, 12 (24%) radiation oncologists, 11 (22%) pulmonologists, 2 (4%) clinical oncologists and 1 (2%) thoracic surgeon.

Most of respondents’ practice involved the care of patients with metastatic cancer: for 15 (30%) of respondents this involved most of their practice, whilst for 34 (68%) a substantial proportion of their practice.

### Availability of SPC services

Forty-eight respondents (96%) had access to at least one component of SPC services, whilst 27 (54%) had access to comprehensive SPC services. See results in Table 1.

**Table 1:** **SPC services available**

Type of service	Service available (n)	%
Hospital based PC teams	39	78
Outpatient/community based PC teams	41	82
Inpatient hospice	34	68
All	27	54
None	2	4

### Referral practices to PC

Participants were asked as to their general practice in relation to PC referrals. The majority of responders (72-76%) refer patients to PC when they have difficult to control symptoms or when no more treatment options are available, but only 12-20% of respondents refer all patients or all symptomatic patients to SPC. All responses are presented in Table 2.

**Table 2:** **Referral practices to SPC**

Referral to PC specialist	Yes (n)	%
Every patient	6	12
Every symptomatic patient	10	20
Patient with symptoms difficult to control	38	76
Patient with no more treatment options to PC team	25	50
Patient with no more treatment options to hospice directly	11	22

### Referral practices according to point in the Disease Trajectory

Participants were asked as to their referrals to PC at different times in the disease trajectory. Only 12%-22% of participants refer almost all or most of their patients to SPC at diagnosis of metastatic disease and during oncological treatment. This increases to 75% when no further oncological treatment is possible and at the end of life. Results are presented in Table 3.

**Table 3:** **Referral to PC according to point on the disease trajectory**

	Almost all	Most	Sometimes/often	Rarely	Never
At diagnosis	4.17%	8.33%	16.67%	64.58%	6.25%
During oncological treatment	8.16%	14.29%	36.73%	36.73%	4.08%
When no further treament available	39.58%	35.42%	18.75%	6.25%	0%
At end of life	58.33%	14.58%	18.75%	8.33%	0%

### Barriers for referral to SPC

Participants were asked to rate on a Likert scale of 0 to 10 (0 = “would not be an issue for me” and 10 = “a very significant issue for me”) about the degree to which six (6) different statements/barriers affect their referral to PC. Subsequently the results were grouped as follows: 0–1 strongly disagree, 2–4 disagree, 5 neutral, 6–8 agree, 9–10 strongly agree. Results are presented in Table 4.

**Table 4:** **Barriers for referral to SPC**

	Strongly disagree	Disagree	Neutral	Agree	Strongly agree
1. Palliative Care physicians are not available in my region/hospital	58%	18%	4%	14%	6%
2. Appointments with palliative care physicians are hard to get	58%	18%	4%	16%	4%
3. My patients do not like being referred to palliative care.	24%	36%	14%	24%	2%
4. Referring to palliative care physicians means that I abandon my patient	54%	34%	4%	2%	6%
5. Palliative care specialists discourage active oncological therapy	46%	40%	6%	8%	0%
6. Palliative care specialists in my country are not experienced/trained enough. I can provide better symptom control management than them.	60%	20%	2%	12%	6%

Availability of PC physicians is a barrier for referral for 20% of respondents. The biggest barrier, expressed by 26% of respondents was that that their patients ‘do not like being referred to PC’. Only 8% of respondents felt that referral to PC signifies abandoning their patients, and that PC specialists interfere or discourage active oncological therapy. Finally only 18% of respondents had concerns about the expertise of their PC colleagues.

### Attitudes regarding SPC

Participants were asked to rate on a Likert scale of 0 to 10, the degree to which they feel SPC can help with the following eight issues. Answers were subsequently grouped as follows: 0–1 strongly disagree, 2–4 disagree, 5 neutral, 6–8 agree, 9–10 strongly agree. Results are presented in Table 5.

**Table 5:** **Attitudes towards SPC**

SPC can help with:	Strongly disagree	Disagree	Neutral	Agree	Strongly agree
1. Patients physical, psychological and spiritual symptoms	12%	6%	8%	50%	24%
2. Enhance quality of life and positively influence the course of illness	12%	4%	2%	66%	16%
3. Caregivers education/support and help them deal with anxiety and distress	14%	8%	10%	56%	12%
4. Provide respite care for caregivers	12%	10%	22%	46%	10%
5. Improve patients’ illness understanding	18%	20%	10%	42%	10%
6. Difficult communication issues (end of life discussions)	18%	8%	8%	50%	16%
7. Provide end of life care	12%	0%	2%	52%	34%
8. All of the above	38%	0%	4%	50%	8%

For most issues regarding SPC, the majority of respondents had positive attitudes, with only 12-26% negative and 2-10% neutral views. However, regarding SPC’s role to improve patients’ illness understanding and help with all the above (questions 5 and 8), there was more disagreement with 38% of respondents expressing negative views and 4-10% being neutral.

Analysis was undertaken to examine whether there was a correlation of responses (referral patterns and attitudes of participants) according to services available to them. Respondents were grouped into: group A: having full comprehensive (i.e. all) SPC services available to them, B: Some SPC services and C: no SPC services available to them. Respondents with comprehensive SPC services available to them (group A) were more likely to refer patients to SPC for patients with difficult to control symptoms, but their practice did not differ significantly from the other respondents, with less comprehensive SPC services available to them(group B and C), in relation to referring all patients or all symptomatic patients, or patients with no more treatment options (Table 6). Regarding attitudes of respondents towards SPC, according to availability of SPC services to them, there were no statistically significant differences.

**Table 6:** **Referral patterns according to SPC services available**

Referral to SPC according to available SPC Services available				
	Palliative care services available			
	All (Hospital based PC teams, outpatient, inpatient) (N = 27)	Some (N = 21)	None (N = 2)	P-value exact test
	N (%)	N (%)	N (%)	
**Palliative care referral for all patients**				
**No**	23 (85.2)	19 (90.5)	2 (100.0)	0.7552
**Yes**	4 (14.8)	2 (9.5)	0 (0.0)	
**Palliative care referral for symptomatic patients**				
**No**	20 (74.1)	18 (85.7)	2 (100.0)	0.5735
**Yes**	7 (25.9)	3 (14.3)	0 (0.0)	
**Palliative care referral for all patients with diffiuclt to control symptoms**				
**No**	3 (11.1)	7 (33.3)	2 (100.0)	0.0060
**Yes**	24 (88.9)	14 (66.7)	0 (0.0)	
**Palliative care referral for outpatient review if no more treatment options available**				
**No**	15 (55.6)	8 (38.1)	2 (100.0)	0.1676
**Yes**	12 (44.4)	13 (61.9)	0 (0.0)	
**Palliative care referral directly to the hospice if no more treatment options available**				
**No**	19 (70.4)	18 (85.7)	2 (100.0)	0.3331
**Yes**	8 (29.6)	3 (14.3)	0 (0.0)	

Finally seventy-eight (78%) of LC specialists expressed an interest to participate in a trial randomizing EPC for patients with metastatic lung cancer.

## Discussion

The response rate to this survey (29.4%) although low is similar to the ESMO survey [6] (34.4%) and the Australian surveys by Ward [7] (30.3%) and Johnson [8] (48%), although clearly lower than the Canadian survey by Wendtland [10] (72%), where both emailed and mailed invitations for participation were sent, and where a small financial gift was also offered. In our survey a total of three (3) emails were sent, and potentially a higher response rate may have been achieved by sending also mailed invitations and providing a small financial gift/voucher.

There was good availability of SPC services in centres where EORTC LCG members work, with almost all (96%) participants having access to at least one component of SPC services, whilst more than half (54%) had access to comprehensive SPC services. Similar data were seen in the recent Canadian survey by Wendtland [10], where 94% of oncologists had access to at least one component of an SPC service, and 36% had access to comprehensive services, and the Australian survey by Ward [7], where 96.5% of Australian medical oncologists reported access to a SPC service. In contrast this differs from the ESMO survey [6] undertaken almost 10 years ago, where only a minority of European Medical Oncologists collaborated often with a PC care specialist (35%), a palliative home care service (38%) or an in-patient hospice (26%). This difference may reflect both an improvement of availability of SPC services in European countries over the last 10 years, and the fact that members of the EORTC Lung Cancer Group work in large, academic centres more likely to have SPC services. The availability of SPC in Europe, Australia and Canada, is clearly better than in most countries worldwide including the US [11], where less than 60% of National Cancer Institute (NCI) Cancer Centres have access to outpatient SPC, whilst in smaller non NCI Cancer Centres such access is only 22% according to data published in 2010 and 2011 [12]. The availability of SPC in the developing world is clearly a much bigger problem [13].

In terms of referral patterns of the EORTC Lung Cancer members to SPC, there is a clear pattern of increasing referrals towards the end of life. This was very consistent, as when asked in two different ways (questions 5 and 6), only 10-12% of respondents would refer their patients to SPC at diagnosis of metastatic disease, whilst when no further oncological treatment is possible and at the end of life, between 72-76% of EORTC Lung cancer specialists would refer almost all or most of their patients to SPC (Tables 2 and 3). The conclusion is therefore that whilst EORTC LCG members refer the majority of their patients to SPC, most of these patients are in fact referred late in the disease trajectory. This is very similar to the practice of Canadian Oncologists [10]; 84% of them stated that they refer terminally ill patients usually/always, and only one third refer at the diagnosis of metastatic disease, with only 13% referring at a prognosis of greater than 6 months [10]. This however needs to be contrasted to the at least theoretical preference of Australian Oncologists for a concurrent rather than a sequential model of care, with 51.3% expressing a preference for concurrent management introduced as complex care needs increase and 39.1% throughout the entire course of advanced cancer [7], and the agreement that early referral is beneficial (71%), and that patients may benefit from SPC services while still receiving disease-modifying therapies (92%) [8].

The timing of referral to SPC is particularly important, i.e. the issue of early PC (EPC) referral, given the benefits seen in terms of survival, quality of life and depression in the study by Temel et al [3] and the ASCO PCO advocating EPC for all patients with advanced cancer [4], i.e. a concurrent model of delivery of active oncological and PC. The alternative approach of a sequential model of care, and hence a late referral to SPC may result in physical, psychosocial and spiritual-religious symptoms and needs remaining unattended or potentially ineffectively managed for longer. Late referrals also result in relatively little time for SPC providers to deal with these symptoms so close to the end of life, hence less likely to be effective in addressing these symptoms. Furthermore late referral to PC, results in PC often being viewed as end of life care, resulting in negative preconceptions towards PC, which is the underlying reason for patients and relatives refusal to be referred for PC. Hence another benefit from this early referral to PC and a concurrent PC and oncological care approach, is that it does away with the negative preconceptions and stigma of PC being viewed as end of life care, allowing for a smoother introduction to PC, without ‘abandonment’ by the oncologist [7].

The biggest barrier for referral to SPC in this survey was negative attitudes of patients to PC (cited by 26% of participants). It should be noted that this reflects the respondents’ perception of this, and not the actual attitudes of patients. Other barriers for referral to SPC in this survey were the lack of availability of PC services (20%), lack of expertise of PC physicians (18%), and finally 8% of participants felt that referral to PC signifies abandoning their patients, and that PC specialists interfere or discourage active oncological therapy. Similarly in both the Australian survey by Ward [7] and the Canadian study [10] the biggest barrier for referral related to negative attitudes of patients to PC, but at a much higher rate than in our survey. In the Canadian study [10], 43% of oncologists felt that their patients had a negative perception of the term palliative care, whilst in the study by Ward, “reluctance for referral” was reported by almost 70% of patients (minor 60.9%, major 8.7%) and even higher by families (minor 67%, major 7%). Other significant barriers in this survey related to availability of SPC services: a lack of inpatient beds (minor 27%, major 34.8%) and inadequate resources for specialist palliative care to take some referrals (minor 30.4%, major 30.4%)” [7]. In the other Australian study by Johnson [8], the main barrier for referral was the perception that the cancer specialist could manage the symptoms (64.2%); whilst also higher than in our study was the rate of specialists feeling of abandoning the patient (19.8%) [8]. Hence whilst similar barriers were expressed in all the other surveys [6–8, 10], these were reported at a much higher rate than in this survey. This may reflect the increased need of Lung Cancer (LC) patients for PC in view of the heavy symptom burden and poor prognosis associated with LC, and possibly a greater familiarity of LC specialists with SPC services.

The fact that the biggest barrier to referral appears to be the negative attitudes of patients towards PC, suggests that indeed there are negative preconceptions among the public towards PC, possibly due to an association with Hospice Care and a perception that this is end of life Care, “where people go to die”. In support of this, comes the finding from the Canadian survey that one third of Canadian Oncologists would be more likely to refer to SPC earlier if it was renamed supportive care [10]. Two studies from the M. D. Anderson Cancer Center also showed that renaming PC to supportive care may increase SPC referrals or facilitate earlier referrals. Fadul et al [14] in a survey of 100 medical oncologists and 100 midlevel providers found that more participants preferred the name supportive care compared with palliative care (57% vs 19%, p < 0.0001), and that participants expressed an increased likelihood to refer patients on active primary treatment (79 vs 45%, p < 0.0001) and advanced cancer treatment (89 vs 69%, p < 0.0001) if PC was renamed supportive care. The name PC compared with supportive care was perceived more frequently as a barrier to referral, decreasing hope and causing distress in patients and families [14]. In a subsequent study from the same group, following a name change for the service from PC to supportive care, an increase in both inpatient referrals and earlier referrals in the outpatient setting was found [15].

A previous review by Ahmed et al [16], looking at barriers to access and referral to PC, identified lack of knowledge and education amongst health and social care professionals, and a lack of standardized referral criteria as key factors. It has also been suggested that unfamiliarity of oncologists with SPC services, resulting in oncologists not being aware of the potential impact of SPC to meet the complex needs of patients with advanced cancer, as well as perceptions of some oncologists, that either nothing can be done about these issues or that they can do all this by themselves, may be to blame [17]. This has major consequences for both Oncological and PC services, and the realization of a common goal of providing better quality patient-centred services. To address this unfamiliarity of Oncologists with PC and SPC services, it is vital that clinical training programs in PC are made compulsory for all oncologists (both trainees as well as practicing oncologists) [18]. In fact in the Canadian study by Wendtlandt et al., it was found that “controlling for age and specialty, those who completed a rotation in palliative care during their residency were twice as likely to refer before chemotherapy started than during chemotherapy, corroborating previous evidence that experience with SPC may influence referral practices” [10]. Hence providing PC training for oncologists could improve patient care both directly by better symptom control as a result of better PC skills of oncologists (oncologists providing basic PC) and indirectly by increasing referrals to SPC services.

The majority of Lung Cancer specialists in this survey expressed positive attitudes towards PC. There were however up to 22% of participants who had overtly negative attitudes and this was similar to the ESMO survey, where 15% had pervasively negative views [6].

The strengths of this study relate to the fact that it provides more updated information regarding oncologists’ attitudes and referral patterns to SPC compared to the previous important studies by Cherny et al [6], the two Australian surveys by Ward et al [7] and by Johnson et al [8], and the Canadian Oncologists survey by Wendtland [10], which were undertaken prior to the Temel study [3] and the ASCO PCO [4]. In addition in our study the views of LC specialists were sought, to examine more specifically the impact of the Temel study and the ASCO PCO on their practice regarding referrals to PC. We can conclude that as yet LC Specialists in Europe have not adopted an EPC concurrent with active oncological care approach. A possible explanation for this, may be that whilst it appears that there is good availability of SPC services in European centres, LC specialists may be either concerned that these SPC services are currently fully utilized with limited capacity to cope with more and earlier PC referrals, or by conviction avoid to refer early to PC, unless absolutely necessary e.g. due to extremely difficult to control symptoms (Table 6). It may also be that LC specialists are not yet fully aware of, or are not convinced of the benefits of EPC, which certainly applies for the 22% minority of respondents with negative attitudes towards SPC. Finally what may underlie this reluctance to adopt EPC is the question regarding the reproducibility of the Temel study, in other health care settings outside the US. This is in view of concerns that the benefits seen with the Temel study may relate to the relatively under-developed SPC services in the US [11], and the different models of SPC provision in the US compared to Australia, Canada and the UK. In the US until now PC was considered to be synonymous with hospice care and hence an option when patients entered the terminal phase of their illness [7], although there is a major effort to change this, e.g. with the ASCO PCO [4]. In contrast in Canada, Australia, the UK and some other European countries shared models of care with concurrent PC and oncological care are more common. There is therefore a need for further studies to provide more evidence regarding the potential benefits of the concurrent approach of EPC and SOC. The majority of respondents in this survey expressed an interest to participate in a European, multi-centre, multi-national study of EPC.

Limitations of this survey include the low response rate, which although similar to the previous Australian and European surveys [6–8], is just under 30%. There is therefore a potential response bias (which applies to all of the previous surveys as well) that responders were more likely to be more familiar or in favour of referring to SPC services, than those that failed to participate in this survey, and hence their answers may reflect a more pro-PC view. Furthermore the members of the EORTC Lung Cancer Group surveyed, are more likely to work in large European tertiary Oncology Centres, and likely to have better access to PC services than Oncologists working in smaller Oncology centres. Finally there is a potential response bias due to LC specialists being aware of the Temel study and ASCO guidelines, providing more ideal answers than what their actual practice is. All these three factors could potentially bias the survey towards providing replies of earlier referral to SPC. However given that the actual data in this survey show that referrals are made late during the disease trajectory, even within the context of good availability of SPC services, within large academic centres and potentially even with LC specialists respondents more interested in PC, this would give further support to the findings of the study i.e. that a concurrent model of care has not been adopted and that the recent guidelines have not been translated into clinical practice for LC patients in Europe.

## Conclusions

This survey of European Lung Cancer (LC) specialists found good availability of SPC services in the centres where European LC specialists work. The majority of LC specialists had positive attitudes towards SPC, whilst the biggest barrier to referral was their perception that patients do not like being referred to PC. In terms of their referral patterns, LC Specialists refer most patients to SPC, but predominantly late in the disease trajectory, hence the practice of Early PC (EPC) concurrent with standard oncological care has not been adopted. Further research in concurrent models of care, with PC delivered concurrently with anticancer treatment, is warranted to provide more evidence regarding the benefits of this approach.

## Electronic supplementary material

Additional file 1: **Questionnaire to EORTC Lung Cancer Group members.** (DOCX 15 KB)

## References

[1] Peppercorn JM, Smith TJ, Helft PR, Debono DJ, Berry SR, Wollins DS, Hayes DM, Von Roenn JH, Schnipper LE (2011). American society of clinical oncology statement: toward individualized care for patients with advanced cancer. J Clin Oncol.

[2] WHO (1990). 11 Technical Report Series. Cancer Pain Relief and Palliative Care.

[3] Temel JS, Greer JA, Muzikansky A, Gallagher ER, Admane S, Jackson VA, Dahlin CM, Blinderman CD, Jacobsen J, Pirl WF, Billings JA, Lynch TJ (2010). Early palliative care for patients with metastatic non-small-cell lung cancer. N Engl J Med.

[4] Smith TJ, Temin S, Alesi ER, Abernethy AP, Balboni TA, Basch EM, Ferrell BR, Loscalzo M, Meier DE, Paice JA, Peppercorn JM, Somerfield M, Stovall E, Von Roenn JH (2012). American society of clinical oncology provisional clinical opinion: the integration of palliative care into standard oncology care. J Clin Oncol.

[5] SAS Institute Inc (2014). SAS® 9.4 Statements: Reference, Third Edition.

[6] Cherny NI, Catane R, European Society of Medical Oncology Taskforce on Palliative and Supportive Care (2003). Attitudes of medical oncologists toward palliative care for patients with advanced and incurable cancer: report on a survey by the European Society of Medical Oncology Taskforce on Palliative and Supportive Care. Cancer.

[7] Ward AM, Agar M, Koczwara B (2009). Collaborating or co-existing: a survey of attitudes of medical oncologists toward specialist palliative care. Palliat Med.

[8] Johnson CE, Girgis A, Paul CL (2008). Cancer Specialists’ palliative care referral practices and perceptions: results of a national survey. Palliat Med.

[9] Breuer B, Fleishman SB, Cruciani RA, Portenoy RK (2011). Medical oncologists’ attitudes and practice in cancer pain management: a national survey. J Clin Oncol.

[10] Wentlandt K, Krzyzanowska MK, Swami N, Rodin GM, Le LW, Zimmermann C (2012). Referral practices of oncologists to specialized palliative care. J Clin Oncol.

[11] Morrison RS, Augustin R, Souvanna P, Meier DE (2011). America's care of serious illness: a state-by-state report card on access to palliative care in our nation's hospitals. J Palliat Med.

[12] Hui D, Elsayem A, De la Cruz M, Berger A, Zhukovsky DS, Palla S, Evans A, Fadul N, Palmer JL, Bruera E (2010). Avaliability and integration of palliative care at US Cancer Centres. JAMA.

[13] Webster R, Lacey J, Quine S (2007). Palliative care: a public health priority in developing countries. J Public Health Policy.

[14] Fadul N, Elsayem A, Palmer JL, Del Fabbro E, Swint K, Li Z, Poulter V, Bruera E (2009). Supportive versus palliative care: what's in a name? a survey of medical oncologists and midlevel providers at a comprehensive cancer center. Cancer.

[15] Dalal S, Palla S, Hui D, Nguyen L, Chacko R, Li Z, Fadul N, Scott C, Thornton V, Coldman B, Amin Y, Bruera E (2011). Association between a name change from palliative to supportive care and the timing of patient referrals at a comprehensive cancer center. Oncologist.

[16] Ahmed N, Bestall JC, Ahmedzai SH, Payne SA, Clark D, Noble B (2004). Systematic review of the problems and issues of accessing specialist palliative care by patients, carers and health and social care professionals. Palliat Med.

[17] Abrahm JL (2012). Integrating palliative care into comprehensive cancer care. J Natl Compr Canc Netw.

[18] Charalambous H, Silbermann M (2012). Clinically based palliative care training is needed urgently for all oncologists. J Clin Oncol.

[CR19] The pre-publication history for this paper can be accessed here:http://www.biomedcentral.com/1472-684X/13/59/prepub

