# Supplementary material for: Attitudes and referral patterns of lung cancer specialists in Europe to Specialized Palliative Care (SPC) and the practice of Early Palliative Care (EPC)
Source: BMC Palliat Care. 2014 Dec 16;13:59. doi: 10.1186/1472-684X-13-59 (PMC4279692; doi:10.1186/1472-684X-13-59)
Supplement: Supplementary file 1 — Additional file 1: Questionnaire to EORTC Lung Cancer Group members. (DOCX 15 KB) [file 12904_2014_236_MOESM1_ESM.docx]

**Additional file 1. Questionnaire to EORTC Lung Cancer Group members:**

1. Country where you practice:
2. What is your specialty: medical oncology / radiation oncology / pulmonology / surgery / other: please specify ………………………………………
3. How much of your practice involves the care of patients with metastatic (incurable) cancer?
   1. None
   2. Small proportion
   3. A substantial proportion
   4. Most of my practice
4. Which palliative care services are available to your patients?
   1. Hospital based palliative care teams Yes / No
   2. Outpatient / Community based palliative care teams Yes / No
   3. Inpatient Hospice Yes / No
   4. None
   5. Other, please specify:……………………………………………………………………………….
5. Please comment (you can mark more than one) as to the palliative care practice where you work with respect to patients diagnosed with advanced (not radically treatable) lung cancer. Please tick what applies:
   1. Every patient has a one to one interview with either a palliative care consultant or specialist palliative care nurse (or lung cancer specialist nurse with palliative care training)
   2. Every symptomatic patient has a one to one interview with either a palliative care consultant or specialist palliative care nurse (or lung cancer specialist nurse with palliative care training)
   3. If a patient has symptoms that are difficult to control they are referred to the palliative care consultant or specialist palliative care nurse.
   4. If a patient has no more treatment options they are referred to the palliative care consultant or specialist palliative care nurse.
   5. If a patient has no more treatment options they are referred direct to the hospice.
   6. If none of the above statements describes the practice at your institution, please provide details: …………………………………………………………………………………………………………………………..
6. How often do you refer your metastatic lung cancer patients to palliative care?

|  | At diagnosis of metastatic disease | During their oncology treatment | When no further treatment possible | For end of life / hospice care |
| --- | --- | --- | --- | --- |
| Almost all (>75%) |  |  |  |  |
| Most (50-75%) |  |  |  |  |
| Often (25-50%) |  |  |  |  |
| Rarely (<25%) |  |  |  |  |
| Never |  |  |  |  |

1. Please comment as to factors / barriers for referring to palliative care.

| Please rank each of the following on a scale of 0 to 10 where 0 is “would not be an issue for me” and 10 is “a very significant issue for me” (higher numbers indicate more significant issue) | |
| --- | --- |
| a. Palliative Care physicians are not available in my region / hospital |  |
| b. Appointments with palliative care physicians are hard to get |  |
| c. My patients do not like being referred to palliative care. |  |
| d. Referring to palliative care physicians means that I abandon my patient |  |
| e. Palliative care specialists discourage active oncological therapy |  |
| f. Palliative care specialists in my country are not experienced / trained enough. I can provide better symptom control management than them. |  |

1. In your opinion specialist palliative care can help with:

| Please rank each of the following on a scale of 0 to 10 where 0 is “would not be an issue for me” and 10 is “a very significant issue for me” (higher numbers indicate more significant issue) | |
| --- | --- |
| - 1. patients physical, psychological and spiritual symptoms |  |
| - 1. enhance quality of life and positively influence the course of illness |  |
| - 1. difficult communication issues (end of life discussions) |  |
| - 1. improve patients’ illness understanding |  |
| - 1. caregivers education/support and help them deal with anxiety and distress |  |
| - 1. provide respite care for caregivers |  |
| - 1. provide end of life care |  |
| - 1. all of the above |  |

1. Would you be interested to participate in a trial randomizing into a formal introduction of early palliative care for your patients with metastatic lung cancer? Yes / No
